# Distinct signals of clinal and seasonal allele frequency change at eQTLs in *Drosophila melanogaster*

**DOI:** 10.1111/evo.14617

**Published:** 2022-09-20

**Authors:** Yang Yu, Alan O. Bergland

**Affiliations:** ^1^ Department of Biology University of Virginia Charlottesville Virginia 22904

**Keywords:** Clinal adaptation, eQTL, expression, seasonal adaptation

## Abstract

Populations of short‐lived organisms can respond to spatial and temporal environmental heterogeneity through local adaptation. Local adaptation can be reflected on both phenotypic and genetic levels, and it has been documented in many organisms. Although complex fitness‐related phenotypes have been shown to vary across latitudinal clines and seasons in similar ways in *Drosophila melanogaster* populations, the comparative signals of local adaptation across space and time remain poorly understood. Here, we examined patterns of allele frequency change across a latitudinal cline and between seasons at previously reported expression quantitative trait loci (eQTLs). We divided eQTLs into groups by using differential expression profiles of fly populations collected across latitudinal clines or exposed to different environmental conditions. In general, we find that eQTLs are enriched for clinally varying polymorphisms, and that these eQTLs change in frequency in concordant ways across the cline and in response to starvation and chill‐coma. The enrichment of eQTLs among seasonally varying polymorphisms is more subtle, and the direction of allele frequency change at eQTLs appears to be somewhat idiosyncratic. Taken together, we suggest that clinal adaptation at eQTLs is at least partially distinct from seasonal adaptation.

Identifying the evolutionary forces that maintain genetic variation in natural populations remains one of the key questions in population genetics (Gillespie 1998; Charlesworth and Charlesworth 2017). One strong diversifying force is environmental heterogeneity (Dobzhansky 1955; McDonald and Ayala 1974; Gillespie 1998), which can result in the selective maintenance of genetic variation within and between populations (Levene 1953; Haldane and Jayakar 1963; Gillespie and Turelli 1989; Turelli and Barton 2004; Charlesworth 2006). Environmental change across the range of many widely distributed species is often associated with latitudinal gradients related to phenology (Viegas et al. 2012; Fjellheim et al. 2014; Kong et al. 2019) and spatial adaptation to temperate environments (Bradshaw et al. 2004). For organisms with short generation times, temporal variation in selection pressures can drive adaptive tracking (Botero et al. 2015). Adaptive tracking has been shown to occur in response to seasonal variation in selection pressures (Dobzhansky and Ayala 1973; Mueller et al. 1985; Rodríguez‐Trelles et al. 1996; Ananina et al. 2004; Bergland et al. 2014; Wittmann et al. 2017), and in principle these adaptive fluctuations across seasons should mirror spatial variation because of common selective pressures imposed by seasonality (Singh and Rhomberg 1987).

Empirical work on *Drosophila melanogaster* has shown parallel differentiations in fitness‐related traits across a latitudinal cline and between seasons. Lab‐reared descendants of flies collected in the spring are more starvation tolerant and show a wider breadth of thermal tolerance, similar to lab‐reared descendants of flies collected in northern locales (Schmidt et al. 2005, Schmidt et al. 2008; Schmidt and Paaby 2008; Behrman et al. 2015). Genetic and genomic work has shown that allele frequency shifts between seasons sometimes show parallel clinal variation (Bergland et al. 2014; Cogni et al. 2014; Paaby et al. 2014; Machado et al. 2021). For instance, inversion frequency of *In(3R)Payne* shows a strong latitudinal cline in North America and stable oscillations between seasons at an orchard in Pennsylvania (Kapun et al. 2016). Candidate adaptive polymorphisms affecting diapause in the gene *couch‐potato* show parallel shifts in frequency across space and time: the pro‐diapause allele has higher frequency in the spring and in the north, compared to the fall or the south (Cogni et al. 2014).

Although there is growing evidence of parallelism across latitudinal and seasonal gradients in flies, only a small fraction of clinally and seasonally varying SNPs overlap (∼3.7%; Rodrigues et al. 2021). Such low proportion of overlap could arise from several factors. First, the demographic history of flies collected across a latitudinal cline and between seasons differs (Bergland et al. 2014, Bergland et al. 2016): clinally varying polymorphisms may be a consequence of secondary contact and seasonally varying polymorphisms might be affected by severe overwintering bottlenecks. Second, selective forces that vary across latitudinal clines might not exactly mirror those across seasons. Finally, the causal loci of adaptation across latitudinal clines might be different from adaptation across seasons.

To understand the comparative signals between clinal and seasonal adaptation, we studied the spatial and temporal distribution of alleles associated with genetic variation in gene expression. Gene expression variation has been demonstrated to be important for adaptive evolution in many organisms (King and Wilson 1975; Gompel et al. 2005; Fraser et al. 2010; Richards et al. 2012; Fraser 2013; Mack et al. 2018). As a consequence, loci associated with expression (eQTLs) could show parallel adaptive signals across space and time, and can be used to test hypotheses about local adaptation (Fraser et al. 2011). Knowledge of eQTL identity provides information about the functional significance of noncoding polymorphisms and can therefore be used to provide insight into the function of polymorphisms that vary across space and time. More generally, we can ask whether eQTLs are likely to contribute to rapid spatial and temporal adaptation, and test whether the patterns of allele frequency change are similar at eQTLs between space and time. In addition, knowledge of eQTLs allow us to test hypotheses about the direction of allele frequency change through space and time using information about adaptive differentiation in gene expression (Juneja et al. 2016) and expression plasticity (Zhou et al. 2012).

## Materials and Methods

### POPULATION ALLELE FREQUENCIES AND STATISTICS

An overview of data and analysis is explained in Figure S1. We used allele frequency estimates at ∼1.7M SNPs from 45 samples (Table S1) as reported by Machado et al. (2021). This dataset includes populations sampled along the east coast of North America (“clinal”), and 20 paired spring‐fall samples from geographically distributed localities across two continents (“Core 20”). Two paired spring‐fall samples (BA_12 and VI_12) from the Core20 were mislabeled (Nunez et al. 2021); we corrected their labeling in our analysis. Machado et al. (2021) modeled allele frequency change at each SNP across space and time using generalized linear models. The multipopulation seasonal model used “spring” and “fall” labels as independent variables and the multipopulation clinal model used latitude (hereafter “cross‐population”). We used the output of those models to define “seasonal” and “clinal” polymorphisms based on *P*‐value and regression coefficients. In general, we used *P*‐values for enrichment tests and the regression coefficients representing the direction of allele frequency change across space and time for directionality tests. We also examined the allele frequency change between spring and fall for each of the Core20 population pairs independently, as well as between Florida and Maine samples to characterize differences between the endpoints of our clinal analysis as described in “DIRECTIONALITY ANALYSIS OF eQTL FREQUENCY” section.

### eQTL IDENTITY

Our study used eQTLs identified by Everett et al. (2020) that are also polymorphic among the clinally and seasonally sampled populations. Everett et al. (2020) identified eQTLs using RNASeq data on pre‐genotyped inbred DGRP lines against SNPs with >0.05 allele frequency and <25% missing phenotypes for both sexes (3–5 days mated, whole body). We grouped eQTLs into female‐specific, male‐specific, and non‐sex‐biased based on their association with each of ∼4000 genes and novel transcribed regions, hereafter referred to as “genes.” Of the 104,592 autosomal eQTLs (SNPs) originally identified (Everett et al. 2020), 72,389 were identified as polymorphic among the clinal and seasonal dataset. The high proportion of SNPs shared between the DGRP lines and the wild populations that we study reflects the recent shared evolutionary history of the DGRP and the other North American populations that we use to study spatial and temporal patterns of allele frequency change.

### MATCHED CONTROLS

For enrichment and directionality analyses, we compared eQTLs to sets of matched control SNPs (hereafter “controls”) that were not identified as eQTLs themselves. For each eQTL, we identified 1000 control SNPs matched for chromosomal arm, heterozygosity (binned by 0.05), and inversion status classified as “breakpoint” (±0.5 Mb around known inversion breakpoints), “inside” the inversion region and excluding breakpoint regions, “outside” the inverted region and excluding break regions, of six cosmopolitan inversions (Corbett‐Detig and Hartl 2012; Bergland et al. 2014; Machado et al. 2021). Heterozygosity, ranging from 0 to 0.5 with an increment of 0.05, for each SNP was estimated from the DGRP. These sets of controls are used throughout, unless otherwise noted.

### GENOME‐WIDE ENRICHMENT ANALYSIS

We tested if eQTLs are enriched for clinal or seasonal SNPs relative to controls based on their ranked clinal or seasonal *P*‐value quantiles. For the test set of eQTLs or each of the 1000 sets of control SNPs, we used the counts of SNPs above and below a range of *P*‐value quantiles (0.001–0.5) to calculate 1000 odds ratios. We calculated the odds ratio (OR) as AD/BC, where A and C are the counts of eQTLs (A) or controls (C) below or equal to a certain ranked *P*‐value quantile, and B and D are the counts of eQTLs (B) or controls (D) above a certain ranked *P*‐value quantile. We log_2_‐transformed odds ratio and calculated confidence interval as 1.96 × standard deviation of the mean (1000 sets). In addition, to break linkage among the eQTLs, we randomly sampled one eQTL per 10 kb for 100 times and reperformed the enrichment analysis.

### INVERSION ANALYSIS

To test if eQTLs located inside (∼19.5k), near the breakpoints (∼4.3k), or outside (∼59.6k) of cosmopolitan inversions *In(2L)t*, *In(2R)NS*, *In(3L)P*, *In(3R)K*, *In(3R)P*, and *In(3R)Mo* are enriched for clinal or seasonal SNPs, we partitioned each chromosomal arm into “breakpoint,” “inside,” and “outside” for each inversion separately. We performed the enrichment analysis using top 5% clinal or seasonal *P*‐value quantile for eQTLs, and their matched controls.

### GENE‐SPECIFIC ENRICHMENT ANALYSIS

To determine whether the genome‐wide enrichment signals observed are driven by specific genes, we partitioned the eQTLs (both *cis‐* and *trans‐*) by genes. For each gene, we calculated the proportion of its eQTLs that are in the top 5% of clinal or seasonal SNPs. We calculated the gene‐specific enrichment as an odds ratio (described above) relative to matched controls. We only included genes with at least one eQTL in the top 5% of clinal (1093 genes) or seasonal (1158 genes) *P*‐value quantiles for this analysis.

### DIRECTIONALITY ANALYSIS OF eQTL FREQUENCY

To test whether allele frequency change at eQTLs across space and time matches known patterns of differential expression, we performed a directionality analysis by calculating concordance scores. The concordance score is the fraction of eQTLs or controls that change allele frequency across space or time in the predicted manner. We defined three outcomes from the analysis: (1) Concordant: when concordance score is significantly higher than the null expectation of 50%; (2) Discordant: when concordance score is significantly lower than the null, indicating the opposite directions as expected; (3) Neutral: when the concordance score is not significantly different from the null.

We included two differential expression (DE) datasets for this directionality analysis. One dataset identified genes that show parallel differential expression in females between populations derived from high and low latitudes in Australia and North America and reared in a common environment (Juneja et al. 2016), hereafter referred to as “latitudinal DE genes.” We used female‐specific (*n* = 1392) and non‐sex‐biased (*n* = 880) eQTLs because Juneja et al. (2016) measured differential expression only in females. Of the 159 genes identified by Juneja et al. (2016), we used 39 that overlapped with the eQTL dataset. The second dataset identified genes differentially expressed in response to heat shock (57 genes), chill‐coma (16), starvation (28), high temperature (19), and low temperature (20) among an outbred panel derived from the DGRP (Zhou et al. 2012). We used non‐sex‐biased eQTLs (*n* = 4844 in total) for this dataset.

To calculate concordance scores at eQTLs, we combined the sign of allelic effects at eQTLs (i.e., up‐ or downregulating) with the observed change in gene expression in the two differential expression datasets. For example, for genes with higher expressions in northern populations compared to southern ones, we hypothesized that the eQTLs associated with an increase in gene expression should be more common in northern than southern populations. The converse would be the case for genes that are more highly expressed in southern populations. Fly populations collected in the spring are thought to be more “winter adapted” than those collected in fall (Bergland et al. 2014), and thus we hypothesized that spring‐fall comparisons would mirror north‐south comparisons.

We applied a similar approach to genes expressed in response to several environmental treatments (Zhou et al. 2012). We hypothesized that for genes upregulated following chill‐coma, starvation, or low‐temperature exposure, the associated upregulating eQTL alleles will be more common in the north and in the spring, relative to the south or the fall. Low temperature stands for constant low‐temperature treatment (18°C), whereas chill coma stands for acute 3 h on ice followed by 1‐h recovery treatment (Zhou et al. 2012). Our assumption is that populations in the north or in the spring are more likely to experience both constant low temperature and acute chill shocks because they live in an environment with comparatively low temperature and more likely to encounter chill shock. Conversely, we hypothesized that for genes upregulated following heat‐shock and high‐temperature exposure, the upregulating alleles would be less common in the north and in the spring.

We then calculated concordance scores. We examined directionality based on the cross‐population clinal and seasonal models. To determine whether the cross‐population concordance signals observed are driven by specific genes, we partitioned the eQTLs by genes. For each gene, we calculated concordance score using clinal or seasonal cross‐population models.

In addition, we also examined eQTL directionality between pairs of populations. For the spatial comparison, we compared allele frequencies between Florida and Maine. For the seasonal comparisons, we compared allele frequencies between spring and fall within a sampling locality. The locality‐specific seasonal comparison is meant to assess the consistency of allele frequency change between seasons across populations. We generated expected distributions using 1000 control sets.

### EMPIRICAL *P*‐VALUES

For the enrichment and directionality analysis, we calculated empirical *P*‐values. Let *S* be the observed value and *S*_0_ be the expected distribution generated by 1000 sets of controls, *N* is the total number of tests, and defined

$$P=\left(1+\sum \left(S\geq S_{0}\right)\right)/\left(N+1\right),$$

$$emp.P=2\times \min\left(P,1-P\right)$$

(Davison and Hinkley 1997). For *emp.P* = 0 in our tests, we report *emp.P* < 0.001.

## Results

### GENOME‐WIDE ENRICHMENT TEST

**Figure 1:**
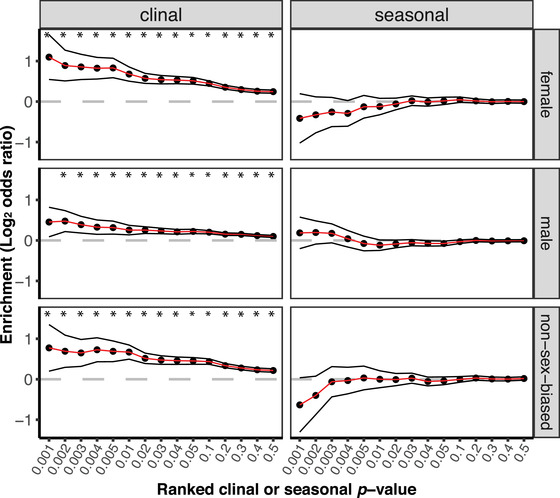
Enrichment of clinal or seasonal SNPs in female, male, and non‐sex‐biased eQTLs genome wide. The *x*‐axis is ranked clinal (left) or seasonal (right) *P*‐value thresholds. The *y*‐axis is enrichment, calculated as the log_2_(odds ratio) of eQTLs having ranked clinal or seasonal *P*‐values below or equal to certain thresholds compared to controls based on matching parameters. Black dots represent average log_2_(odds ratio) over 1000 bootstraps. Black lines are confidence intervals, represented by 1.96 standard deviations of the mean over 1000 bootstraps. Asterisks indicate significant enrichment after Bonferroni correction for 15 tests (empirical *P* ≤ 0.003).

To understand clinal and seasonal allele frequency change at eQTLs, we examined whether eQTLs are enriched for clinal or seasonal SNPs. We find significant enrichment of clinal SNPs in 44 out of 45 of our tests (adjusted *emp.P* ≤ 0.003; Fig. 1; Table S2) but not seasonal SNPs for female, male, and non‐sex‐biased eQTLs across a range of *P*‐value quantiles. To test if this result is affected by linkage disequilibrium, we randomly sampled one eQTL per 10 kb, and again show enrichment of clinal SNPs in female, male, and non‐sex‐biased eQTLs across a range of clinal *P*‐value quantiles (Fig. S2). The decrease in clinal enrichment among the downsampled eQTLs (Figs. 1 vs. S2) suggests that clinal eQTLs are heterogeneously distributed throughout the genome at the most stringent clinal *P*‐value quantiles. However, the general trend of clinal enrichment signals is not solely affected by linkage.

**Figure 2:**
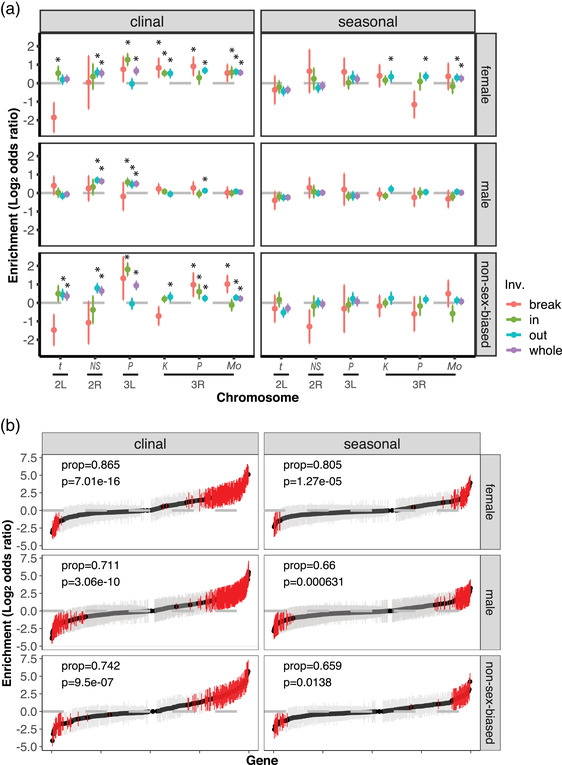
Enrichment of clinal or seasonal SNPs in female, male, and non‐sex‐biased eQTLs on each chromosomal arm (whole) and inversion (breakpoints, inside, outside) regions (a), and in every gene identified with eQTL (b). (a) The *x*‐axis is chromosomal arms. Error bars are confidence intervals, represented by 1.96 standard deviations of the mean over 1000 bootstraps. Asterisks indicate significant enrichment after Bonferroni correction for 22 tests (empirical *P* ≤ 0.002). (b) The *x*‐axis is genes identified with eQTLs. Genes are ranked by averaged log_2_ odds ratio within each analysis type (clinal or seasonal) and sex (female, male, non‐sex‐biased) combination panel. The *y*‐axis is enrichment. Black dots represent average log_2_(odds ratio) over 1000 bootstraps. Red or gray error bars are 1.96 standard deviations of the mean over 1000 bootstraps for genes with significant or insignificant signals, respectively. Proportion (prop) represents the ratio between genes significantly enriched for clinal or seasonal eQTLs and the total number of genes with significant (enrichment and depletion) signals (empirical *P* ≤ 0.05).

Next, we tested whether inversion status of the eQTLs (inside, outside, or near breakpoints of six cosmopolitan inversions) affects clinal or seasonal enrichment signals. We show chromosome‐wide enrichment signals of clinal SNPs in eQTLs on chromosomal arms 2L (non‐sex‐biased: *emp.P* < 0.001), 2R (all: *emp.P* < 0.001), 3L (all: *emp.P* < 0.001), and 3R (female, non‐sex‐biased: *emp.P* < 0.001), and enrichment of seasonal SNPs in eQTLs on chromosomal arm 3R (female‐: *emp.P* < 0.001). eQTLs near inversion breakpoints for *In(3R)P* (female, non‐sex‐biased: *emp.P* < 0.001), *In(3R)Mo* (non‐sex‐biased: *emp.P* < 0.001), and *In(3R)K* (female: *emp.P* < 0.001) or within inverted regions for *In(3L)P* (all: *emp.P* < 0.001), *In(2L)t* (female: *emp.P* < 0.001), *In(3R)P* (non‐sex‐biased: *emp.P* < 0.001), *In(3R)Mo* (female: *emp.P* < 0.001), and *In(3R)K* (female‐: *emp.P* < 0.001) are enriched for clinal SNPs. We do not observe enrichment signals for seasonal SNPs in eQTLs near inversion breakpoints or within inverted regions (Fig. 2a).

To address whether the enrichment signals are driven by a limited number of genes, or by many genes, we performed gene‐specific enrichment analysis. We find that 23.79% and 10.10% of genes included in the analysis are significantly enriched (*emp.P* ≤ 0.05) for clinal and seasonal eQTLs, respectively (Fig. 2b). In addition, there is a strong excess of genes that are significantly enriched for clinal or seasonal eQTLs, compared to genes that are depleted for clinal or seasonal eQTLs, respectively (proportions > 0.5, *P* < 0.05; Fig. 2b). Our results suggest that the genome‐wide enrichment signals are not driven by a small number of genes (Table S3).

### THE DIRECTIONALITY OF eQTL FREQUENCY CHANGE ACROSS SPACE AND TIME

**Figure 3:**
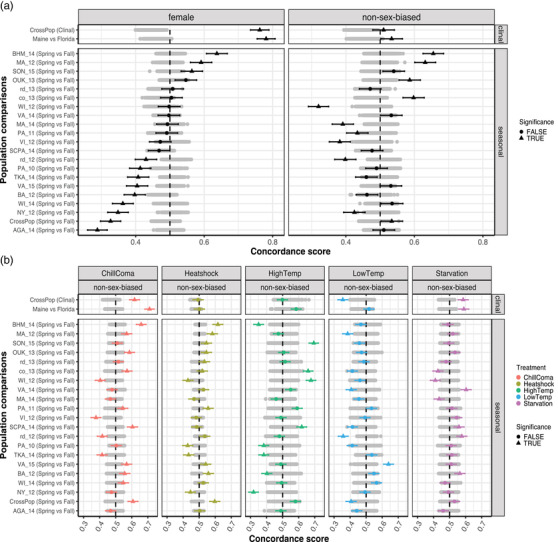
The directionality of allele frequency change for female, non‐sex‐biased eQTLs associated with genes differentially expressed between high and low latitudinal populations (a), or for non‐sex‐biased eQTLs associated with genes differentially expressed under certain environmental treatments (b). The *x*‐axis is the concordance score. The *y*‐axis is clinal or seasonal population comparisons, with the assumption that gene expression patterns are similar between northern and spring populations, and similar between southern and fall populations. The cross‐population comparison results were generated by using clinal or seasonal coefficients, whereas other comparisons used allele frequencies of eQTLs from each sample pair. Black (a) or colored (b) dots (triangles and circles) are observed values of eQTLs. Gray circles are expected distributions generated by control SNPs, bootstrapped 1000 times. Black (a) or colored (b) triangles indicate observed concordance scores at eQTLs significantly deviate from null distributions, with Bonferroni correction of empirical *P*‐values for 23 tests (empirical *P* ≤ 0.002). Black (a) or colored (b) circles indicate nonsignificant deviations of observed eQTLs values from expected control distributions.

We tested whether eQTLs show concordant changes in allele frequency across the cline or between seasons. We show that female, non‐sex‐biased eQTLs associated with latitudinal DE genes are more likely to change allele frequencies between clinal populations in a concordant way than controls (CrossPop: *emp.P* < 0.001; FL‐ME: *emp.P* < 0.001; Fig. 3a). We also show discordant signal for female eQTLs associated with latitudinal DE genes in the seasonal comparison (CrossPop: *emp.P* = 0.002), and concordant change for non‐sex‐biased eQTLs (*emp.P* = 0.002; Fig. 3a). The significant cross‐population signals at latitudinal DE genes are likely driven by *Hsc70‐2* with ∼1000 female eQTLs (Fig. S3a). eQTLs associated with *Hsc70‐2* are strongly concordant across the latitudinal cline (∼80%) but discordant (∼25%) between seasons. The gene‐specific concordance score also varies from gene to gene (Fig. S3). In addition, the eQTLs associated with latitudinal DE genes do not always change allele frequencies in predicted directions in every paired spring‐fall sample, suggesting that seasonal changes in selection pressure might not always be consistent between populations (Fig. 3a).

Next, we evaluated the directionality of eQTL allele frequency change at environmental DE genes (Fig. 3b). Consistent with our predictions, eQTLs associated with DE genes under starvation (CrossPop: *emp.P* < 0.001, FL‐ME: *emp.P* < 0.001) and chill‐coma (CrossPop: *emp.P* < 0.001, FL‐ME: *emp.P* < 0.001) treatments show concordant change in clinal comparisons, suggesting plastic genes induced by these treatments could be adaptive. In contrast to our prediction that northern flies are more “cold adapted,” eQTLs associated with low‐temperature‐treatment‐induced DE genes show discordant signal for clinal comparison (CrossPop: *emp*.*P* = 0.002). In seasonal comparisons, we show concordant signal for eQTLs associated with chill‐coma and heat‐shock (CrossPop: *emp*.*P* < 0.001) induced DE genes, consistent with our prediction that spring flies are more chill‐coma resistant and less heat shock resistant, but discordant signal for eQTLs affecting low‐temperature‐treatment induced DE genes (CrossPop: *emp.P* = 0.002). Like results for eQTLs associated with latitudinal DE genes, the gene‐specific concordance score at environmental DE genes varies from one gene to another (Fig. S3b) and the directionality for eQTLs’ allele frequency is inconsistent among Core20 spring‐fall comparisons (Fig. 3b).

## Discussion

Although there are well‐documented patterns of local adaptation across latitudinal clines and between seasons in *D. melanogaster*, we still have a limited understanding of the genetic architecture of this evolutionary change. Here, we show that the signal of clinal and seasonal adaptations differs at eQTLs, suggesting that distinct evolutionary processes occur across space and time in this species. Our results rely on signals of enrichment and concordance that are calculated across eQTLs relative to controls. One caveat for our analyses is that eQTLs could be linked and less so for control SNPs. Thus, our analyses should be interpreted as a way to identify linked sets of SNPs that are enriched for functionality, as eQTLs, and spatial or temporal adaptive signals. This work provides novel insight into our understanding of spatial and temporal differentiation by identifying loci that are functionally and physically linked.

### ENRICHMENT

Gene expression has been shown as an important driver of local adaptation (López‐Maury et al. 2008; Colicchio et al. 2020). Consistent with the expectation that gene expression variation contributes to clinal adaptation (Adrion et al. 2015), we show eQTLs are enriched for clinal SNPs (Fig. 1). The levels of clinal enrichment observed are comparable to those observed for other functional categories in *Drosophila* (Machado et al. 2016) and other species (Ye et al. 2013; Mack et al. 2018). Such an enrichment agrees with growing evidence that spatial differentiation at eQTLs contributes to local adaptation across taxa (Fraser 2013; Gould et al. 2017; Mack et al. 2018; Phifer‐Rixey et al. 2018; Kitano et al. 2019; Colicchio et al. 2020). We also show that the genome‐wide enrichment signal is not driven by a few genes or solely due to linkage at a single locus (Figs. 2b, S2), suggesting that clinal adaptation at eQTLs is polygenic (Mateo et al. 2018).

We further show evidence that clinal differentiation at inversions may be driven by eQTLs. First, eQTLs near breakpoints or within inverted regions on chromosomal arms 2L, 3L, and 3R are more “clinal” than controls in the same regions (Fig. 2a). This result agrees with previous studies reporting latitudinal inversion clines in North America (Mettler et al. 1977; Kapun et al. 2014). Next, we observe clinal enrichment within inverted regions, but not near breakpoints, on chromosomal arms 2L and 3L (Fig. 2a), suggesting that eQTLs, other than the chromosomal inversions, could be the target of selection. Such a result is consistent with the hypothesis that chromosomal inversions have little effect on gene expression and that natural selection acts on linked loci associated with the inversions, other than the structural variants themselves, in this species (Lavington and Kern 2017; Said et al. 2018). Thus, eQTLs might be the causal driver of allele frequency associated with the inversions on chromosomal arms 2L (Said et al. 2018) and 3L, forming the inversion clines (Kapun et al. 2016).

We hypothesized that seasonal enrichment patterns at eQTLs should mirror clinal patterns (Rhomberg and Singh 1988; Machado et al. 2021). However, we do not observe enrichment of seasonal SNPs at eQTLs, like we do for clinal ones (Figs. 1, 2), either genome wide or at inversions. Genome‐wide, the lack of seasonal enrichment signal could be a result of polygenic adaptation at different alleles due to genetic redundancy (Barghi et al. 2019). For example, if different sets of eQTLs are under seasonal selection pressures in different geographic locations or across years, we might not be able to observe significant overlap between eQTLs and seasonal SNPs identified from the cross‐population model. Alternatively, lack of enrichment could be due to subtle changes of small effect eQTLs that are sufficient for seasonal adaptation collectively, but individually undetectable by the seasonal model.

For eQTLs located near breakpoints or within inversion regions, despite strong clinal enrichment, we do not observe any seasonal enrichment signal (Fig. 2a). Previous studies have argued the importance of inversions underlying seasonal adaptation by showing seasonal inversion frequencies in various drosophilid species (Dobzhansky and Ayala 1973; Knibb 1986; Sanchez‐Refusta et al. 1990) and enrichment of seasonal SNPs at inversion breakpoints or inside inverted regions in *D. melanogaster* genome (Machado et al. 2021). Our result does not contradict such arguments, but rather indicates that previously reported seasonal polymorphisms at these inversions may not be among our analyzed eQTLs (Kapun et al. 2016; Machado et al. 2021). Although the lack of seasonal enrichment patterns could be due to a possible lack of power in detecting seasonal SNPs (e.g., Bergland et al. 2014), it could also indicate that there are ecologically relevant idiosyncratic allele frequency changes between spring and fall among populations (see *eQTL DIRECTIONALITY* section, below).

### eQTL DIRECTIONALITY

Here, we tested whether eQTLs associated with differentially expressed genes show predicted allele frequency change across a latitudinal cline and between seasons. eQTLs associated with previously identified latitudinal DE genes (Juneja et al. 2016) show concordance in allele frequency between Florida and Maine, and across a latitudinal cline in North America in general (Fig. 3a). Such concordant change suggests that these eQTLs are both functional and potentially underlie adaptive differentiation across spatial gradients.

Based on previous work (Bergland et al. 2014; Machado et al. 2021; Rodrigues et al. 2021), we predicted that alleles favored in high‐latitude locales would be more common in the spring compared to the fall. Interestingly, we found the opposite pattern at female eQTLs (Fig. 3a), which is in contrast to previous results (Machado et al. 2021). For the directionality analysis of latitudinal DE genes, our results are strongly driven by a single gene, *Hsc70‐2* (Fig. S3a). This gene is associated with ∼1000 eQTLs that span almost 1 Mb on chromosome 3R, which are likely in long‐distance linkage as a consequence of a partial soft sweep at *Ace* (Garud et al. 2015). Therefore, our results show that the previously reported concordance of allele frequency through space and time (Machado et al. 2021; Rodrigues et al. 2021) is not consistent across the genome and may vary from gene to gene. Such gene‐to‐gene variation in concordance score is also reflected at environmental DE genes (Fig. S3b). Thus, our results demonstrate that considering the inferred functional significance of clinal and seasonal polymorphisms at a gene level is important for interpreting whether shared selection pressures exist between latitudinal and seasonal gradients.

Interestingly, when we applied the directionality test to single‐population spring‐fall comparisons, we show that some populations showed concordant signals, and some showed discordant signals at latitudinal and environmental DE genes. For example, 15% of comparisons show concordance at eQTLs, whereas 40% of comparisons show discordant signals when using eQTLs grouped by latitudinal DE genes (Fig. 3a). Such idiosyncratic directionality patterns among populations are similar to Erickson et al. (2020) that examined diapause associated SNPs. These results suggest that seasonal selection pressures might not always be consistent.

The use of environmental DE genes in our directionality tests is based on two critical assumptions. The first is that expression plasticity is adaptive. Whether this assumption is valid in general is an open question (Ghalambor et al. 2015), and evidence suggests that whether plasticity is adaptive or maladaptive varies between populations (Huang et al. 2022) and among traits (Huang and Agrawal 2016; Mallard et al. 2020). The tests also assume that the environment changes in a specific way through space and time. For instance, a signal of concordance assumes that flies collected in Maine are subject to more bouts of starvation than flies in Florida, and that flies collected in spring are more “cold adapted” than those collected in the fall. Therefore, to interpret a signal of concordance as evidence of adaptation requires plasticity to be adaptive and that the direction of environmental change align with these assumptions. A signal of discordance could reflect maladaptive plasticity or environmental changes that are opposite to our expectations. Our work cannot test whether plasticity is adaptive or maladaptive, but it can shed light on the consistency of selection through space and time.

We propose several possible explanations for the idiosyncratic direction of allele frequency change among the paired spring‐fall comparisons (Fig. 3). First, selection pressures might not be consistent between spring and fall among different populations or years (Erickson et al. 2020; Machado et al. 2021). Therefore, eQTLs affecting a trait that has fitness advantage in one spring‐fall population might not be favored in another, resulting in inconsistent allele frequency changes among populations. Second, seasonal adaptation could be achieved by a subset of common eQTLs via combinations with other population‐specific seasonal loci. It has been shown in previous studies that different combinations of genetic loci could evolve to adapt to the same selective condition while only a limited number of common loci are identified (Barghi et al. 2019). Such possibility could also explain the lack of genome‐wide enrichment signals of seasonal SNPs in eQTLs (see *ENRICHMENT* section, above). Regardless, it is clear that signals of allele frequency change across latitudinal gradients and between seasons are not identical and thus suggest that the genetic architecture of clinal and seasonal adaptation might be different and that environmental changes across space and time might not reflect each other in ways previously identified.

## CONFLICT OF INTEREST

The authors declare no conflict of interest.

## DATA ARCHIVING

Data and scripts are available at Dryad with DOI: https://doi.org/10.5061/dryad.6m905qg32.

Associate Editor: Dr. N. Singh

Handling Editor: Dr. A. McAdam

## Supporting information

Supplemental Figure S1: Explanation chart for data and analysis.

Supplemental Figure S2: Enrichment of clinal or seasonal SNPs in female‐specific, and male‐specific and non‐sex‐biased eQTLs after block sampling.

Supplemental Figure S3: Gene‐specific directionality of eQTLs grouped by latitudinal‐ or treatment‐DE genes.

Supplemental Table 1: Populational information for the 5 clinal and Core20 (seasonal) populations used in our analysis.

Supplemental Table 2: Summary of genome‐wide enrichment analysis for eQTLs.

Supplemental Table 3: Summary of gene‐based enrichment analysis for eQTLs.
