# Supplementary figures and images for: Distinct signals of clinal and seasonal allele frequency change at eQTLs in Drosophila melanogaster
Source: Evolution. 2022 Sep 20;76(11):2758–68. doi: 10.1111/evo.14617 (PMC9710195; doi:10.1111/evo.14617)

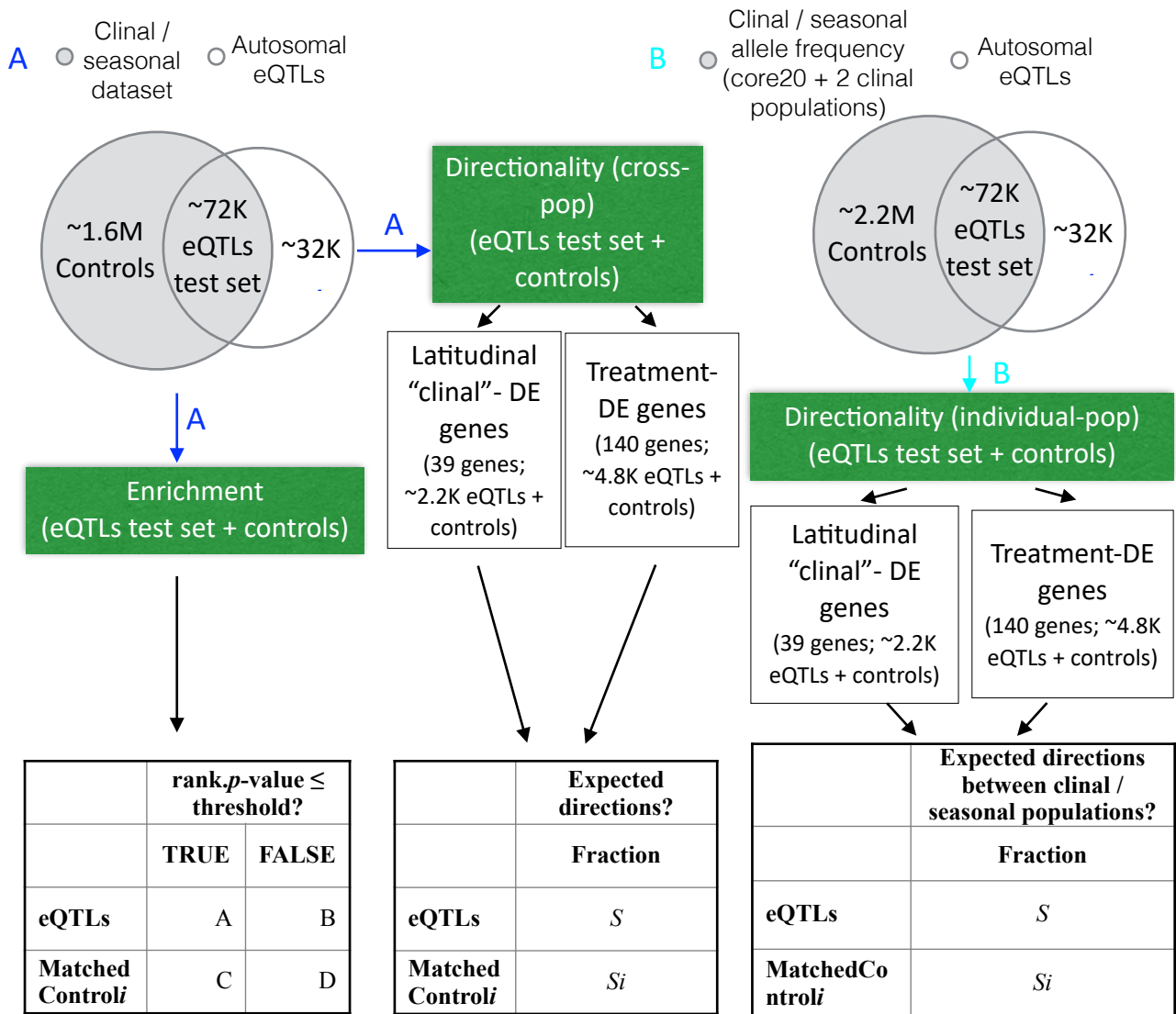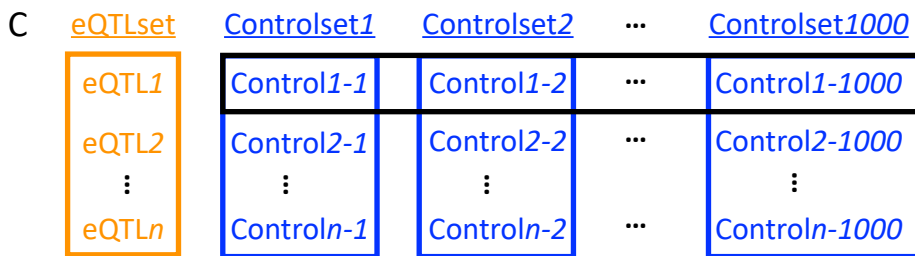

1000 Controls for each eQTL  
1000 Control sets for the eQTL set

Supplement: Supplementary file 1 — Supplemental Figure S1: Explanation chart for data and analysis. [file EVO-76-2758-s003.pdf]

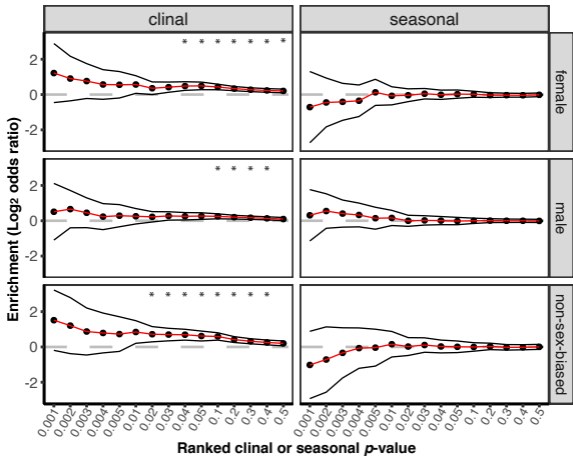

Supplement: Supplementary file 2 — Supplemental Figure S2: Enrichment of clinal or seasonal SNPs in female‐specific, and male‐specific and non‐sex‐biased eQTLs after block sampling. [file EVO-76-2758-s005.pdf]

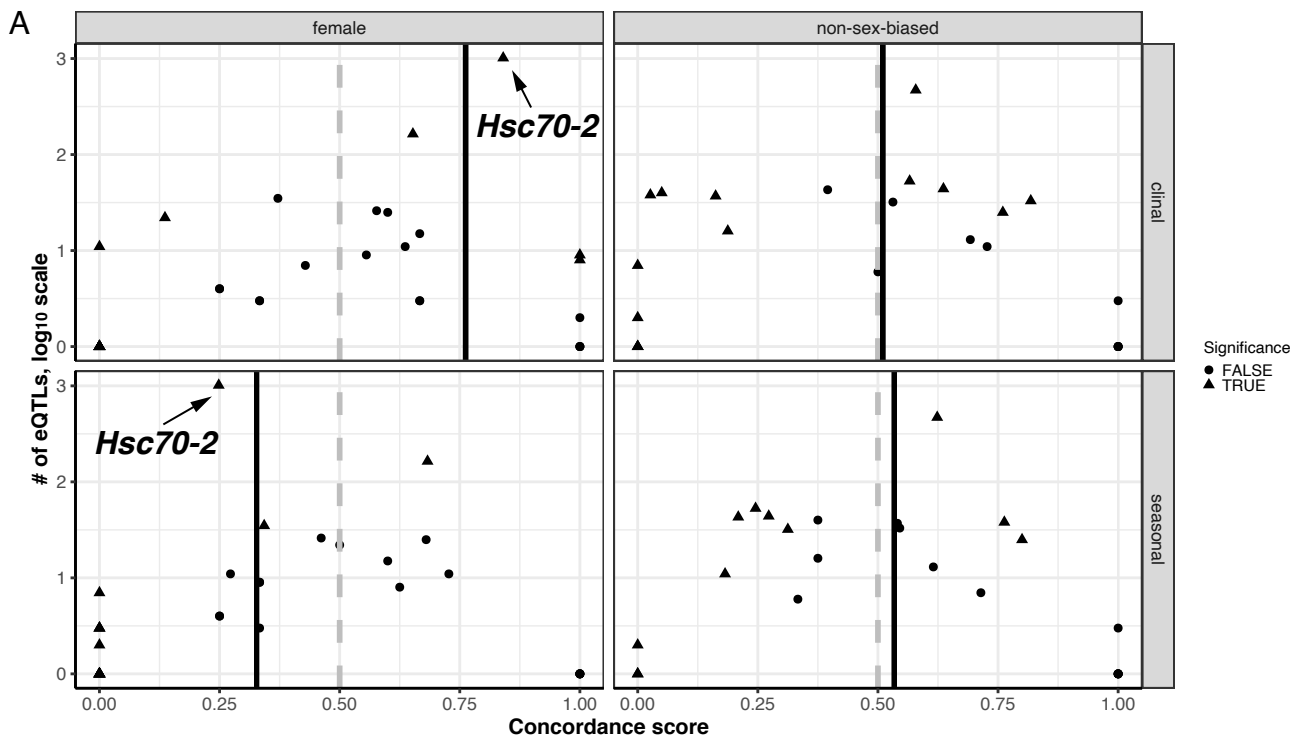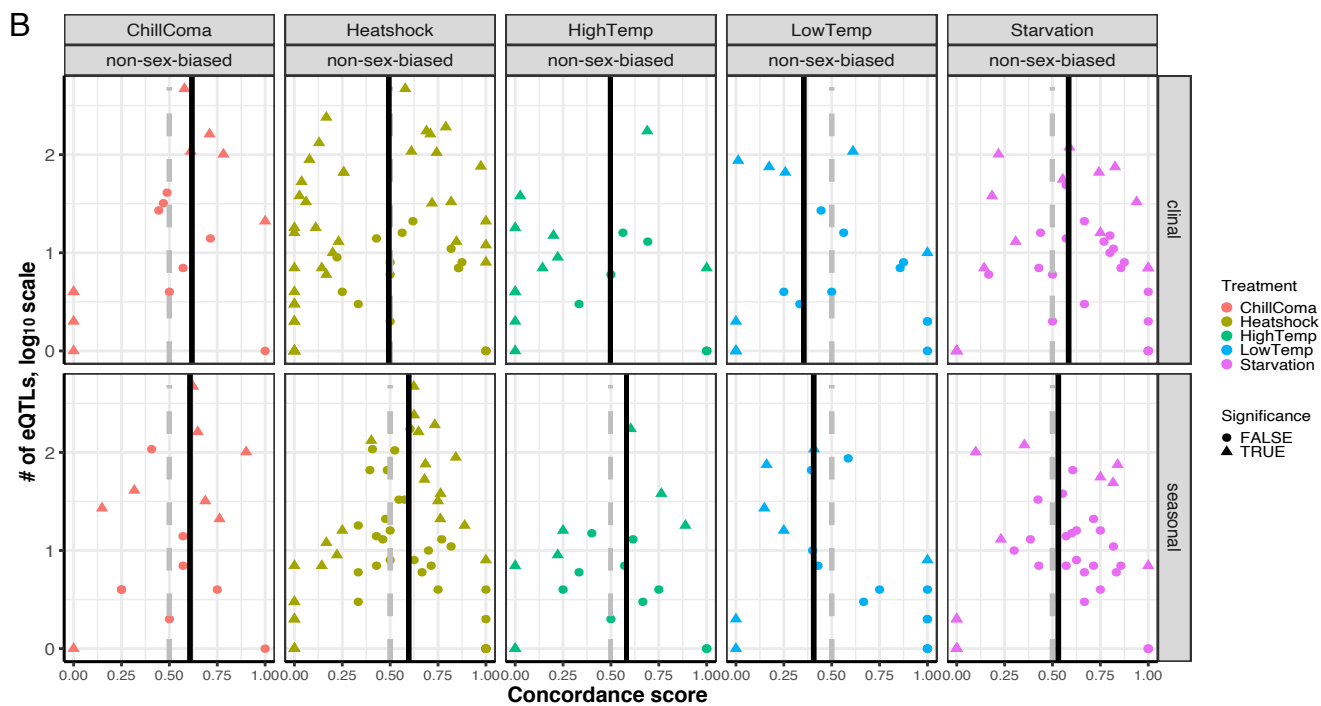

Supplement: Supplementary file 3 — Supplemental Figure S3: Gene‐specific directionality of eQTLs grouped by latitudinal‐ or treatment‐DE genes. [file EVO-76-2758-s002.pdf]
